# Icterus from Insect Bites and Gallbladder Disease

**Published:** 1904-08

**Authors:** 


### Icterus from Insect Bites and Gallbladder Disease.

W. Bauermeister, Brunswick, Germany, (*Therapeutische Monatshefte,* May, 1904,) observes that there not infrequently occurs, as a consequence of the bites of various insects, a more or less extensive urticaria with general dyspeptic symptoms of a gastro-intestinal catarrh. Sometimes yellowness of the scleræ and of the skin demonstrates a duodenal catarrh. Reciprocal action of the skin and the intestinal tract are common phenomena, but the latter is usually the first factor, as in herpes, furunculosis, and similar conditions. In the cases under consideration the trouble emanates in the skin, and is analogous to the occurrence of gastro-duodenal ulcerations after burns, for which the explanation is possibly some physical change in the bile (pleiochromia). Violent irritation of the skin may occasion gastrointestinal ulcerations and lesser disturbances may cause a hyperemic swelling of the intestinal mucosa and a so-called catarrhal icterus. These effects of insect bites the author himself experienced. While visiting a university town in 1901 he was so bitten by bedbugs that he had a severe urticaria lasting two weeks. The attack left him with dyspepsia and so depressed that he had to go to the mountains for recuperation. A violent acute gastroenteritis compelled him to return home. While convalescing therefrom, he was suddenly seized with a gallbladder colic, which recurred at frequent intervals, was accompanied by tension in the region of the liver, and marked swelling of that organ. There was no icterus or marked excess of bile pigment in the urine. The cholecystitis lasted for months, despite the regular use of Carlsbad water.

He then proceeded to a systematic disinfection of the biliary system, as the cholecystitis was dependent upon an infectious catarrh. For this purpose salicylic acid is our most effective remedy; it is in part excreted by the gallbladder walls, the seat of the catarrhal process, and there develops its antiseptic action. An abundant flow of bile is the best natural antiseptic for the biliary passages, and to effect this we have no agents more powerful than salicylic acid and sodium oleate. The author had pills made up containing 1½ grains of each, and later added menthol and phenolphthalein as analeptics and to mildly stimulate intestinal activity. These pills, named probilin, are difficult to prepare, but they are easy to take. In the morning before breakfast and in the evening before retiring he took three or four pills, slowly drinking thereafter about a pint of hot water. After two twenty days' courses of this treatment, with a fortnight's interval between, he gradually mastered the gallbladder infection. He ascribes his recovery entirely to the disinfectant and cholagogue effect of this treatment, and is strengthened in his conviction by the fact that he has succeeded during the past eighteen months in practically curing between sixty and seventy cases of cholelithiasis with and without the passage of gallstones; in only two cases was operation necessary. On the basis of these observations he states that the systematic use of salicylic acid with acid oleate of sodium will help when all other methods, such as Carlsbad with and without rest, eunatrol, turpentine, olive oil, and the like, do no good and the sufferer is face to face with the knife. He details a number of cases.

The treatment is equally efficacious in some cases of bile stasis without concretion formation, i. e., as sometimes observed in cirrhosis of the liver. It appears in the form of a chronic cholangitis, with possibly a slight icterus and irregular intermittent fever and slight chills, general malnutrition and concomitant conditions.

The exact reason for the increased passage of concretions under the probilin treatment is not quite clear. Two halves of a gallstone, equal in size and weight, were immersed in plain water and a probilin solution. The first showed no change after several days, while the second was noticeably smaller and covered with a sticky, slimy coating which the microscope showed to be composed of saponifying cholesterin crystals. The cholesterin rhomboids were softened and polypoid at their edges, showing that they were undergoing solution. The crystals were finally replaced by a mass of softened matter. While this may not occur to the same degree in the gallbladder, it must do so to some extent, since both salicylic acid and soaps are excreted into the viscus. Laboratory reactions also show that not all concretions are equally subject to this solvent action. Bilirubin limestones, pea-sized, examples of which are found not infrequently in the common duct, are recalcitrant to it. And since the salicylic-oleic acid medication acts on these also, its effects cannot be attributed to chemical solution alone.

When the concretion is not simply entangled in the duodenal papillæ, so that it is washed down by the fluid from the stomach, the effect must be due to the increased vis a tergo from the cholagogue action of the remedy, together with the diminished mucosal swelling from the effect of the salicylic acid on the catarrhal process. In point of fact the softness and friability of the calculi found in the gallbladder and ducts when operating on patients who had had probilin, were very noticeable.

In a postscript the author expresses his gratification at the fact that Dr. Kuhn, chief physician in the Elisabeth Hospital at Cassel, a well-known authority in hepatic disease recommends, in the *Berliner Klinik* of June, 1903, salicylic acid and oleate of sodium as the two most reliable drugs. In the Congress of Naturalists at Cassel, Kuhn reiterated his belief in their efficacy and advised the addition thereto of menthol, which is virtually the composition of probilin.

